# Impact of a preoperative pharmaceutical consultation in scheduled orthopedic surgery on admission: a prospective observational study

**DOI:** 10.1186/s12913-020-05623-6

**Published:** 2020-08-13

**Authors:** Amélie Renaudin, Géraldine Leguelinel-Blache, Chloé Choukroun, Audrey Lefauconnier, Christophe Boisson, Jean-Marie Kinowski, Philippe Cuvillon, Hélène Richard

**Affiliations:** 1 Department of Pharmacy, Nimes University Hospital, University of Montpellier, Place du Professeur Robert Debré, 30029 Nîmes Cedex 9, France; 2 UPRES EA2415, Laboratory of Biostatistics, Epidemiology, Clinical Research and Health Economics, Clinical Research University Institute, University of Montpellier, Montpellier, France; 3 Department of Law and Health Economics, Faculty of Pharmacy, University of Montpellier, Montpellier, France; 4 Department of Anesthesia, Nimes University Hospital, University of Montpellier, Nimes, France; 5 Department of Anesthesia, Montpellier Cancer Institute (ICM), University of Montpellier, Montpellier, France

**Keywords:** Pharmacy, Surgery, Anesthesia, Medication errors, Medication reconciliation, Drug safety

## Abstract

**Background:**

Medication errors have a high prevalence in surgery and management of home medication is strongly involved in these errors. In scheduled surgery, the preoperative consultation is a privileged time to inform the patient about the management of her/his home medication before admission. This study assessed the impact of a pre-anesthesia best possible medication history (PA-BPMH) on admission. The PA-BPMH was performed by a clinical pharmacist prior to the anesthesia consultation for anesthesiologists to prescribe admission medical orders for scheduled orthopedic surgery patients.

**Methods:**

This was a prospective observational study which was carried out in an orthopedic surgery department. All patients over 18 years old with an elective orthopedic surgery were eligible except ambulatory surgery patients. The pharmacist registered the PA-BPMH into the software making it available for anesthesiologists for the pre-admission medication order. Finally, a medication reconciliation was performed at admission. The main outcome was the percentage of patients with at least one unintended medication discrepancy (UMD) at admission. The nature, potential clinical impact and acceptance rate of each UMD detected were assessed. Also, the PA-BPMH process was described and patients and anesthesiologists satisfaction was evaluated.

**Results:**

A total of 455 patients had a pharmaceutical consultation. Medication reconciliation was performed at admission for 360 patients. Overall, at least one UMD was observed in 13.0% of patients (*n* = 47). A total of 63 UMD were detected. The most common type of UMD was omission (25.4%) and incorrect drug (23.8%).Two UMD (3.2%) were evaluated as life threatening. All the UMD detected were corrected on the admission medication order.

**Conclusion:**

A preoperative pharmacist-anesthesiologist teamwork seems to improve the safety of perioperative management of home medication for scheduled orthopedic surgery patients. This process needs a randomized clinical trial across a wider range of surgeries before its implementation.

## Background

Adverse drug events (ADE) are a public health problem. Medication management in healthcare institutions has become a major challenge. In fact, ADE occur in 20 to 72% of hospitalizations [1–3] and are responsible for 9.7% of permanent disability [4]. They represent a cost of $2595 to $4685 per ADE [5]. In order to reduce these errors, the medication reconciliation (MR) has been developed. Considered by the World Health Organization important for achieving medication safety, MR is the process of identifying and resolving medication discrepancies to prevent ADE at interfaces of care [6]. The MR is used to compare the best possible medication history (BPMH) and the current admission medication order (AMO) to identify and solve unintended medication discrepancies (UMD) [7, 8].

In surgical departments, the incidence of medication errors is higher than in medical departments (24.3% vs. 43%) [9–11]. Indeed, drug management is complex with multiple actors and several places of prescription over often short periods of inpatient stay, increasing the potential risk of medication errors [12]. The roles of each actor, surgeons and anesthesiologists, should be defined for the medication order and the perioperative management of home medication in wards.

In scheduled surgery, the most common medication error found is the omission of home medication [13–16]. In their study, Haley et al. found that 60.3% of patients had at least one medication error related to usual treatment and 43.3% of these errors were considered potentially harmful [17]. In Nîmes orthopaedic surgery department, anesthesiologists prescribe medicines to scheduled patients during the anesthesia consultation. Nevertheless, a three-month study showed that 53% of patients (*n* = 215) had at least one UMD detected at admission by a clinical pharmacist (unpuplished data: preliminary study).

Thus, a pre-anesthesia best possible medication history (PA-BPMH) established by a pharmacist prior to the anesthesia consultation has been implemented in Nimes orthopaedic surgery department. It is available for anesthesiologists for the pre-admission medication order. Indeed, only with an accurate medication history can decisions be made safely regarding the perioperative management of medications.

The primary objective of our study was to assess the impact of PA-BPMH performed by a clinical pharmacist prior to the anesthesia consultation of scheduled orthopedic surgery patients on the number of UMD at admission. Secondary objectives were to characterize UMD and describe the PA-BPMH process.

## Methods

### Study population

This was a prospective observational single centre study which was carried out in a French university hospital in an orthopedic surgery department. The study was performed from June 2018 to February 2019. All patients over 18 years old with an elective orthopedic surgery were eligible. We excluded ambulatory surgery patients and those who already had an anesthesia consultation in another hospital. All methods were carried out in accordance with relevant guidelines and regulations. Informed consent was obtained from all subjects and/or their legal guardians. This study was a practice assessment, so verbal informed consent was obtained from all subjects and/or their legal guardians. According to French law, an Institutional review board (IRB) (number (N°18.07.01) was granted by the Ethics Committee of the Nimes hospital center.

### Study design

Around 1 month before surgery, patients had an appointment for a pharmaceutical consultation 20 min before the anesthesia consultation. The pharmacist established a pre-anesthesia best possible medication history (PA-BPMH) available for the anesthesiologist. During their consultation, anesthesiologists prescribed medication orders using PA-BPMH.

**Fig. 1:**
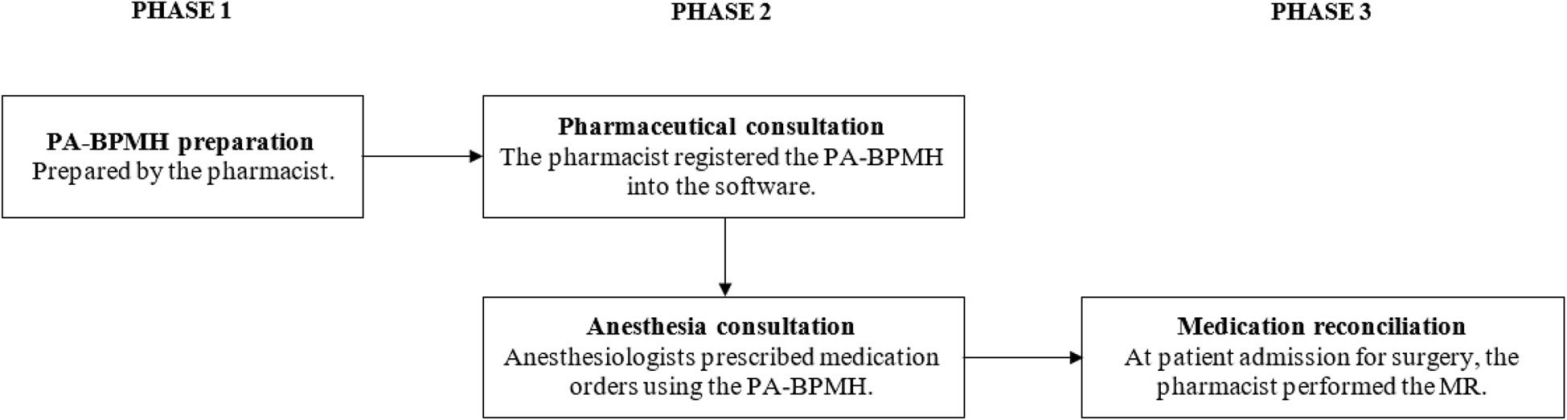
Study design. PA-BPMH: pre-anesthesia best possible medication history, MR: medication reconciliation

The study design was carried out in 3 phases described below in Fig. 1.

#### Phase 1: pharmaceutical consultation preparation

One week before consultation, the pharmacist prepared the PA-BPMH using the electronic hospital scheduling system. First, the pharmacist checked all medical information in the patient medical records and called the community pharmacy who transmitted medication orders. The pharmacist then transcribed them into the hospital prescription support software.

#### Phase 2: pharmaceutical consultation

During consultation, the pharmacist checked all medication data collected with the patient and made proposals for therapeutic equivalences if needed. The pharmacist used the ‘Vitale’ microchip patient card, when available. This card contains a pharmaceutical record fulling from the community pharmacist with dispensed medication history during the last 4 months. The pharmacist registered the PA-BPMH into the software making it available for anesthesiologists for the pre-admission medication order.

The patient satisfaction was evaluated by anonymous self-assessment survey after each pharmaceutical consultation. At the end of the study, the satisfaction of each anesthesiologist using the PA-BPMH was also collected.

#### Phase 3: medication reconciliation at admission

At patient admission, to avoid contamination bias, a medication reconciliation was performed within 24 h by a different clinical pharmacist than the one at the pre-admission phase. The pharmacist established a BPMH and compared it with patient’s admission medication order. He detected and resolved UMD with the physician.

### Outcome measures

The primary outcome was to assess the percentage of patients conciliated at admission with at least one UMD.

Among the secondary outcomes, the nature, potential clinical impact and acceptance rate of each UMD detected were assessed. For clinical impact, methodology used was blind retrospectively evaluation by a senior clinical pharmacist (different from investigators) and a senior anesthesiologist by using the method described by Quélennec et al. [18], a European adaptation of the National Coordinating Council for Medication Error Reporting and Prevention (NCC MERP) Index [19].

Other secondary outcomes were: (1) the time spent at preparation (phase 1) and pharmaceutical consultation (phase 2); (2) type and number of sources for performing PA-BPMH; (3) the rate of therapeutic equivalences proposed by the clinical pharmacist; (4) the satisfaction assessment of patients and anesthesiologists.

For each patient with a complete process (pharmaceutical consultation and medication reconciliation), basic demographical data was collected (age, gender, type of surgery, length of hospital stay) as well as the time between pharmaceutical consultation and patient admission.

### Statistical analysis

Descriptive statistics were reported as counts and percentages for categorical variables, means and standard deviations for continuous variables with normal distribution, and median and quartiles for others. All data collected were entered into REDCap® software (V8.5.1, 2018) [20] and all statistical analyses were performed using Microsoft Excel® software (V2016).

## Results

### Patient characteristics

**Fig. 2:**
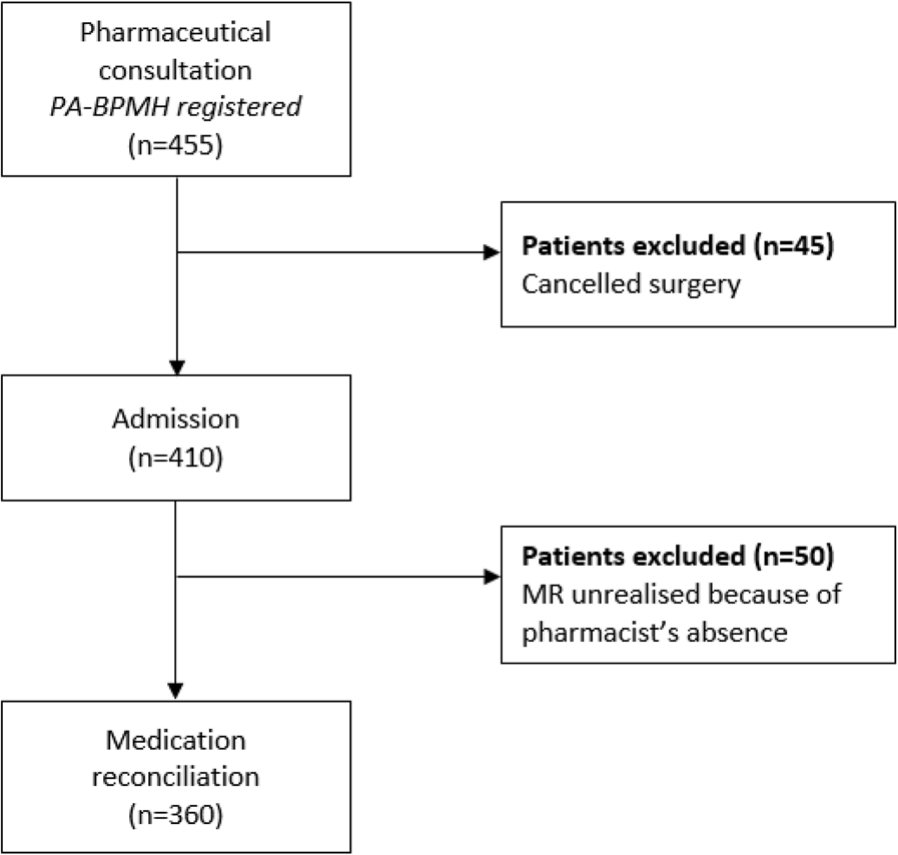
Flow chart. PA-BPMH: pre-anesthesia best possible medication history, MR: medication reconciliation

During the study period, 455 patients had a pharmaceutical consultation. Thereafter, medication reconciliation was performed at admission for 360 patients (Fig. 2). Among these patients, the median age was 68.0 years old (IQR = [59.0; 75.0.]) and 54.0% were female (Table 1). The majority of the study population (83.4%) had home medication and the average number of pre-admission medications was 4.4 ± 3.7 per patient. The median time between the anesthesia consultation and surgery admission was 27 days (IQR = [20.0; 30.0.])

**Table 1:** Baseline characteristics of the study population

Characteristics	Patients (*n* = 360)
Age (yr)	68 [59.0; 75.0]
Sex	
Female	193 (54%)
Male	167 (46%)
Surgical indication	
Knee arthroplasty	141 (39.2%)
Hip arthroplasty	108 (30.0%)
Spine surgery	33 (9.1%)
Other surgery	78 (21.7%)
Number of lines of PA-BPMH	4 [1.0; 7.0]
Length of stay (days)	4 [4.0; 6.0]

### Characterisation of UMD

Medication reconciliation was performed within 24 h after admission for 87.8% of patients (n = 360) who had a pharmaceutical consultation. At least one UMD was observed in 13.0% of patients (*n* = 47). All the UMD detected were corrected on the AMO.

The pharmacist detected 63 UMD, with a mean of 1.3 ± 0.7 UMD per patient. Among these 47 patients, 74.5% had one UMD, 19.1% had 2 UMD and 6.4% had 3 to 5 UMD. The most common type of UMD was omission (25.4%) and incorrect drug (23.8%) or dose (23.8%) (Table 2). The most frequent medications involved belonged to the cardiovascular system (34.9%) (Table 2), including anticoagulants (9.1%), angiotensin-converting enzyme inhibitors (13.6%) and angiotensin receptor blockers (18.2%).

**Table 2:** Unintentional medication discrepancies grouped according to ATC and type of discrepancy

ATC	Omission	Incorrect dose	Incorrect drug	No indication	Incorrect frequency	Total UMD
C	4 (25.0%)	4 (26.6%)	5 (33.3%)	4 (36.4%)	5 (83.3%)	22 (34.9%)
N	2 (12.5%)	7 (46.7%)	4 (26.7%)	3 (27.2%)	0 (0.0%)	16 (25.4%)
R	2 (12.5%)	0 (0.0%)	3 (20%)	2 (18.2%)	0 (0.0%)	7 (11.1%)
J	4 (25.0%)	1 (6.7%)	1 (6.7%)	0 (0.0%)	0 (0.0%)	6 (9.5%)
A	2 (12.5%)	1 (6.7%)	0 (0.0%)	2 (18.2%)	0 (0.0%)	5 (8.0%)
Others	2 (12.5%)	2 (13.3%)	2 (13.3%)	0 (0.0%)	1 (16.7%)	7 (11.1%)
Total UMD	16 (25.4%)	15 (23.8%)	15 (23.8%)	11 (17.5%)	6 (9.5%)	63

Discrepancies were classified into three categories: level 1 no potential harm (NCC MERP category C), level 2 monitoring or intervention potentially required to preclude harm (NCC MERP category D) and level 3 potential harm (NCC MERP categories D and above) (16, 17).

Among the 63 UMD, 38.1% (*n* = 24) errors were classified as level 1, 30.2% (*n* = 19) as level 2 and 31.7% (*n* = 20) as level 3. Two UMD (3.2%) were life threatening involving an anticoagulant and an antiarrhythmic.

### Pharmaceutical consultation process

The median time to prepare PA-BPMH (phase 1) was 17.0 min (IQR = [12.0; 21.0]) and for the consultation itself (phase 2) was 6.0 min (IQR = [4.0;11.0]). The main information sources for performing PA-BPMH during phase 1 and phase 2 were: patient (100%), medical record (100%), community pharmacist (94.8%) and pharmaceutical record (57.1%). The median number of information sources was 4.0 (IQR = [4.0; 5.0]). Also, to optimize medication management, when the home medication was unavailable at the hospital, the pharmacist proposed 268 (12.1%) therapeutic equivalences on 2221 lines of PA-BPMH.

### Patient and anesthesiologist satisfaction

Overall, 45.7% of patient surveys were filled in (*n* = 455). All patients found the pharmaceutical consultation useful and were able to better understand their medication management at the hospital. Also, the pharmacist’s welcome was considered very satisfactory by 92.3% of them.

All anesthesiologists (12 physicians) completed the survey and all found that the pharmaceutical consultation was helpful for their activity, improved the management of home medication safety, ensured better perioperative medication management, and saved time. They all believed the pharmaceutical consultation must be continued.

## Discussion

This study assessed the impact of PA-BPMH on the number of UMD at admission. The PA-BPMH was performed by a clinical pharmacist prior to the anesthesia consultation for anesthesiologists to prescribe admission medical orders for scheduled orthopedic surgery patients.

At least one UMD was observed in 13.0% of patients and all corrected by physicians at admission. Previous studies have shown that medication discrepancies at admission can be decreased with the help of a clinical pharmacist before patient admission [14, 21, 22]. Indeed, without this help, studies estimate that 48.8 to 60.3% of patients have at least one UMD at surgery admission [17, 23, 24]. These results are consistent with our previous study that found 53% of patient had at least one UMD without PA-BPMH.

Among the strengths of our study, to our knowledge, our process is the first model of a clinical pharmacist-anesthesiologist teamwork consulting in the same unit of place and time. This collaboration optimizes the management of home medication safety for scheduled orthopedic surgery patients. Indeed, the PA-BPMH typically takes the pharmacist 23 min and they cross-reference the patient reported information with four information sources to ensure an accurate and complete medication history. The pharmacists seems to be the ideal actors to establish the PA-BPMH because of their focus on medication and their knowledge about it [14, 15]. Also, this pilot study had sufficient patients included (*n* = 360), as well as the observational period (8 months) to ensure its routine implementation is feasible.

Among the UMD, the most common was medication omission as identified in previous studies [22–25]. These UMD were related with medication additions or modifications by other physicians (mostly antihypertensive medication) occurring between anesthesiologist consultation and admission, for example, when patients have the cardiology consultation during this period. These medication modifications are unavoidable and require that any change to the home medication must be checked at admission.

In addition, by placing a pharmacist upstream in the care process, others benefits are expected. Our team of anesthesiologists found that the pharmaceutical consultation was useful, permitting a better perioperative medication management and saving time during their consultation. Also, the pharmacist was very welcomed by most of patients who found the pharmaceutical consultation useful to better understand their home medication management at admission.

The software is a key point of our process. It has permitted that the pharmacist to enter the PA-BPMH making it available for anesthesiologists for the pre-admission medication order. Adequate information technology avoids the errors of transcription order and preserves the veracity of the original information. In fact, for Orser et al [26]*,* with information technology, it is possible to share information and it facilitates coordination between health professionals.

Our study has some limitations. To strengthen the study methodology, a prospective analysis using randomized selection into control and intervention groups should be performed. Tested in scheduled orthopedic surgery, the impact of a pharmaceutical consultation should be extended to other surgical specialties to corroborate our results. In addition, the effectiveness of this activity should be assessed to provide arguments for decision-makers to implement this pharmaceutical consultation. It is also relevant to assess the impact of this model of a clinical pharmacist-anesthesiologist teamwork on clinical patients’ outcomes such as mortality, readmission and ADE occurrence.

## Conclusion

The preoperative pharmaceutical consultation in scheduled orthopedic surgery combined with the anesthesiologist in a teamwork seems to have a positive impact on reduction of unintended medication discrepancies. Further testing of this process across a wider range of surgeries is needed before it can be widely implemented and so we have designed a randomized clinical trial for that purpose (Clinical Trial PREVEMCA NCT04013061). Implementing a pharmaceutical consultation prior to the anesthesia consultation for all scheduled surgeries should be a safe way to reduce significantly the rate of medication errors.

## Data Availability

The datasets used and/or analysed during the current study are available from the corresponding author on reasonable request.

## Abbreviations

ADE: Adverse drug events
AMO: Admission medication order
ATC: Anatomical therapeutic chemical classification system
BPMH: Best possible medication history
IRB: Institutional review board
MR: Medication reconciliation
NCC MERP: National Coordinating Council for Medication Error Reporting and Prevention
PA-BPMH: Pre-anesthesia best possible medication history
UMD: Unintended medication discrepancy
